# Exploring the active site of the *Streptococcus pneumoniae* topoisomerase IV–DNA cleavage complex with novel 7,8-bridged fluoroquinolones

**DOI:** 10.1098/rsob.160157

**Published:** 2016-09-21

**Authors:** Ivan Laponogov, Xiao-Su Pan, Dennis A. Veselkov, Ryan T. Cirz, Allan Wagman, Heinz E. Moser, L. Mark Fisher, Mark R. Sanderson

**Affiliations:** 1 Randall Division of Cell and Molecular Biophysics, King's College, Guy's Campus, London Bridge, London SE1 1UL, UK; 2 Molecular and Clinical Sciences Research Institute, St George's, University of London, Cranmer Terrace, London SW17 0RE, UK; 3 Achaogen, 7000 Shoreline Ct. No. 371, San Francisco, CA 94080, USA

**Keywords:** topoisomerases, protein–DNA complexes, drug design, X-ray crystallography, DNA topological experiments, MIC determinants

## Abstract

As part of a programme of synthesizing and investigating the biological properties of new fluoroquinolone antibacterials and their targeting of topoisomerase IV from *Streptococcus pneumoniae*, we have solved the X-ray structure of the complexes of two new 7,8-bridged fluoroquinolones (with restricted C7 group rotation favouring tight binding) in complex with the topoisomerase IV from *S. pneumoniae* and an 18-base-pair DNA binding site—the E-site—found by our DNA mapping studies to bind drug strongly in the presence of topoisomerase IV (Leo *et al.* 2005 *J. Biol. Chem.* **280**, 14 252–14 263, doi:10.1074/jbc.M500156200). Although the degree of antibiotic resistance towards fluoroquinolones is much lower than that of β-lactams and a range of ribosome-bound antibiotics, there is a pressing need to increase the diversity of members of this successful clinically used class of drugs. The quinolone moiety of the new 7,8-bridged agents ACHN-245 and ACHN-454 binds similarly to that of clinafloxocin, levofloxacin, moxifloxacin and trovofloxacin but the cyclic scaffold offers the possibility of chemical modification to produce interactions with other topoisomerase residues at the active site.

## 1. Introduction

**Figure 1.**
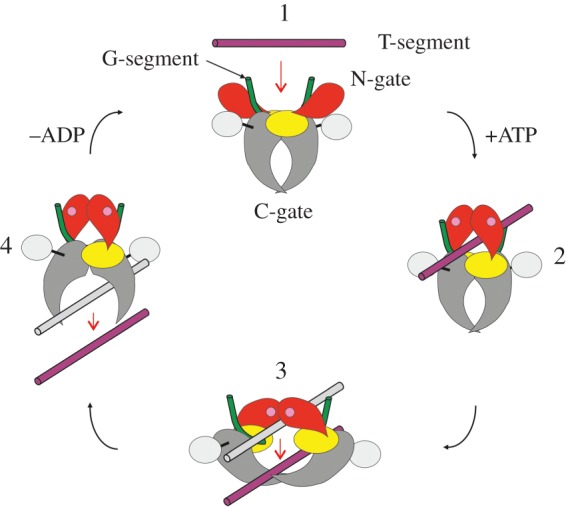
Schematic representation of the catalytic cycle of type II topoisomerases. ParC N-terminal domain (ParC55) is in grey, ParC C-terminal β-pinwheel domain is in silver, ParE N-terminal ATPase domain is in red, ParE C-terminal domain (ParE30) is in yellow, G-gate DNA is in green, transported (T) segment DNA is in purple. Bound ATP is indicated by pink circles in the ATPase domains (reproduced with permission from fig. 1 of [3]).

Quinolone agents, which target type II topoisomerases in Gram-negative and Gram-positive bacteria, are very important drugs in our armoury for the treatment of serious microbial infections and for which there is increasing resistance to β-lactam antibiotics. Quinolone drugs are not exempt from resistance, but the levels of resistance exhibited by this class are at present lower than those for β-lactam-based antibiotics and macrolides (statistics from Canadian Bacterial Surveillance, 2009 [1]). Hence, there is a need to develop effective rationally designed new pharmaceuticals of this class in order to bypass resistance mutations. We have developed 7,8-bridged fluoroquinolones with the aim that the restricted movement of the C7 group should favour tight binding. Quinolones act on type II topoisomerase–DNA complexes to prevent strand passage of the T-segment through the cleaved G-gate in the topoisomerase cycle [2]. When not interrupted by drug interaction, this cycle starts with the reversible binding (through the catalytic tyrosines) of the G-segment DNA to the G-gate consisting of the TOPRIM and WHD domains of the core complex of the type II topoisomerase (figure 1). Capture of a transported T-segment DNA duplex by closure of the N-gate ATPase domains allows strand passage through the double-stranded break in the G-gate. Religation of the cleaved G-DNA segment and exit of the T-DNA duplex through the C-gate completes the passage of one DNA through the other, and after ATP hydrolysis, the enzyme resets for another cycle (figure 1). Quinolones trap the covalent enzyme–DNA cleavage complex, preventing DNA resealing and triggering cell death.

In prokaryotes, DNA topology is controlled by two type II topoisomerase paralogues, topo IV (a tetramer with subunits ParE_2_ParC_2_) and gyrase (a tetramer with subunits GyrA_2_GyrB_2_). The former mediates decatenation of replicated DNA molecules and the latter regulates the overall level of DNA supercoiling within the cell, which is perturbed by polymerases, helicases and other enzymes involved in replication, transcription and recombination [2–14]. The elucidation of the structures of topoisomerase II–drug–DNA complexes at a resolution of 3.5 Å and above by ourselves [15–17] and other workers in the field has meant that there is now a large number of structures deposited with the PDB and a body of knowledge of how a wide range of quinolone and other drugs semi-intercalate into the DNA. How members of the spiropyrimidinetrione compounds [18–20] and also how quinazolinedione compounds [16,21] make contact with the DNA bases and surrounding amino acids residues of their topoisomerase IIA binding sites has been revealed [21–26]. Recent findings of Chan *et al*. show how the spiropyrimidinetrione analogues and etoposide bind differently in the hemi-intercalated binding site in the gyrase complex [23]. We [16,27] and more recently other groups [21,28] have investigated quinazolinediones, a class of antimicrobials that bind through different side-chain interactions to quinolones and select different gyrase and topoisomerase IV mutations in the potential binding volume. Apparently minimal changes in drug structure lead to a lack of cross-resistance with quinolones, an encouraging outcome that can be rationalized structurally and potentially optimized for drug development.

**Figure 2.**
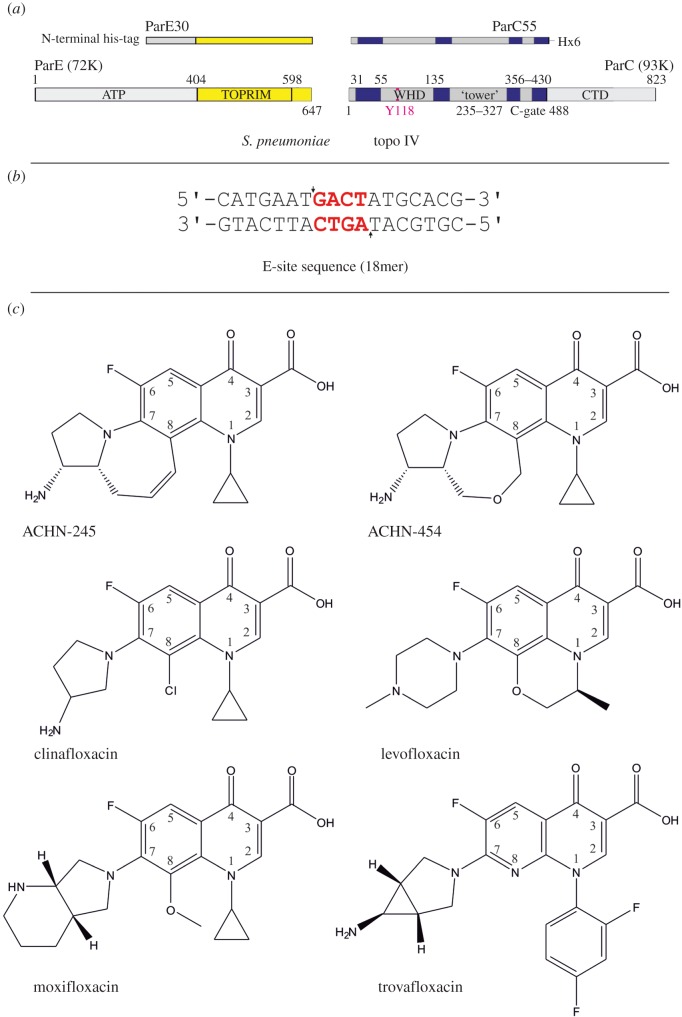
(*a*) Domain composition of the cleavage complexes of topoisomerase IV from *Streptococcus pneumoniae* (top) relative to the full-length proteins ParC and ParE (bottom). (*b*) E-site 18mer DNA sequence; cleavage positions indicated by arrows, 4 bp overhang is highlighted in red. (*c*) Chemical structures of the Achaogen quinolones used in the complexes, ACHN-245 and ACHN-454; chemical structures of the fluoroquinolones clinafloxacin, levofloxacin, moxifloxacin and trovafloxacin, with their respective numbering schemes.

A number of Gram-positive and Gram-negative pathogens have been identified by the Centers for Disease Control, USA as serious antimicrobial resistance threats, of which *Streptococcus pneumoniae* is a major medical concern. *Streptococcus pneumoniae* is a Gram-positive bacterium and an important human pathogen that causes a range of infections, including pulmonary pneumonia, meningitis and otitis. Certain strains have developed resistance to beta-lactams and erythromycin. As part of our ongoing structural and biochemical studies aimed at new topoisomerase-targeting therapeutics, we have focused on topoisomerase IV–DNA complexes with ACHN-245 and ACHN-454, two novel 7,8-bridged fluoroquinolones developed and produced by Achaogen (figure 2*c*). For comparison, figure 2 also shows structures of levofloxacin and moxifloxacin, two clinically important anti-pneumococcal fluoroquinolones, and of the potent investigational quinolones clinafloxacin and trovafloxacin. The new compounds are similar to clinafloxacin in having cyclopropyl and 3-aminopyrroin-1-yl groups at the respective 1- and 7-positions of the quinolone ring (figure 2). However, they differ in having a heptacyclic ring system formed between the quinolone 8-position and the 2-position of the 3-aminopyrrolidin-1-yl substituent at position 7; this seven-membered ring contains a double bond in ACHN-245 and a cyclic ether in ACHN-454. Levofloxacin has, by contrast, a hexacyclic ring system between positions 1 and 8 of the quinolone (figure 2). The attraction of incorporating these bridging ring systems is that they constrain rotation and provide a scaffold for drug engineering, two features that potentially increase binding affinity and selectivity. Here, we describe the enzyme inhibitory and microbiological activities of the compounds. Moreover, we report the structures of the topoisomerase IV–DNA cleavage complexes with ACHN-245 and ACHN-454 at 3.43 and 3.24 Å resolution, respectively.

As in previous structures, we have made use of a DNA duplex corresponding to the E-site, which we originally identified as an *S. pneumoniae* chromosomal DNA sequence that is strongly cleaved by *S. pneunomiae* topo IV (and gyrase) in the presence of a variety of different fluoroquinolones [29,30]. This property makes it an ideal DNA partner for crystallographic work on new quinolones. In contrast with the fluoroquinolone complexes previously published by our group, the E-site DNA oligomers used in this study were changed from 34mers to 18mers (figure 2*b*), as part of an optimization of experimental conditions which has yielded reliable crystallization and structure solution of DNA cleavage complexes. The shorter DNA substrate also produced a change in the crystal space group from the previously reported P3_2_ to P3_1_21 and has allowed higher-resolution structures of the complexes with quinolones to be determined via co-crystallization compared with those obtained by soaking. In turn, it has yielded better-resolved electron density for the chelated magnesium ions, which mediate key interactions among protein, DNA and quinolones in the topoisomerase IV cleavage complex [17,31].

## 2. Material and methods

### 2.1. Cloning, expression and purification of *Streptococcus pneumoniae* ParC and ParE proteins

The cloning, expression and purification protocols for *S. pneumoniae* topoisomerase IV ParC, ParE, ParC55 and ParE30 proteins have been described previously [16,32]. Quinolone-resistant ParC S79F and quinazolinedione-resistant ParE E475A proteins were overexpressed and purified as described [27,33,34].

### 2.2. Preparation of the DNA substrate

In order to form the *S. pneumoniae* cleavage complex, E-site 18mer DNA oligomers (5'-CATGAATGACTATGCACG-3', 5'-CGTGCATAGTCATTCATG-3') were synthesized by solid-phase phosphoramidite chemistry and doubly HPLC purified by Metabion, Munich. The lyophilized DNA oligomers were reconstituted in the annealing buffer (20 mM Tris-HCl, pH 7.5, 200 mM NaCl, 5 mM β-mercaptoethanol, 0.05% NaN_3_), mixed in 1 : 1 molar ratio, heated to 95°C and then allowed to cool slowly to 4°C over a period of 48 h in a Dewar (sealed thermos flask). Macromolecule information is provided in table 1.

**Table 1.** Macromolecule production information. Engineered tags and mutations are underlined.

source organism	*Streptococcus pneumoniae* (isolate 7785, St. George's Hospital; [34])
expression vector	pET19b (N-terminal His_10_), pET29a (C-terminal His_6_)
expression host	*E.coli* BL21(λDE3) pLysS
complete amino acid sequence of the recombinant protein	ParC55: MSNIQNMSLEDIMGERFGRYSKYIIQDRALPDIRDGLKPVQRRILYSMNKDSN TFDKSYRKSAKSVGNIMGNFHPHGDSSIYDAMVRMSQNWKNREILVEMHG NNGSMDGDPPAAMRYTEARLSEIAGYLLQDIEKKTVPFAWNFDDTEKEPTV LPAAFPNLLVNGSTGISAGYATDIPPHNLAEVIDAAVYMIDHPTAKIDKLMEF LPGPDFPTGAIIQGRDEIKKAYETGKGRVVVRSKTEIEKLKGGKEQIVITEIPYE INKANLVKKIDDVRVNNKVAGIAEVRDESDRDGLRIAIELKKDANTELVLNY LFKYTDLQINYNFNMVAIDNFTPRQVGIVPILSSYIAHRREVILARSRFDKEKA EKRLHIVEGLIRVISILDEVIALIRASENKADAKENLKVSYDFTEEQAEAIVTLQ LYRLTNTDVVVLQEEEAELREKIAMLAAIIGDERTMYNLMKKELREVKKKF ATPRLSSLEDTAKALEHHHHHH ParE30: MGHHHHHHHHHHSSGHIDDDDKHMKNKKDKGLLSGKLTPAQSKNPAKNEL YLVEGDSAGGSAKQGRDRKFQAILPLRGKVINTAKAKMADILKNEEINTMIY TIGAGVGADFSIEDANYDKIIIMTDADTDGAHIQTLLLTFFYRYMRPLVEAGH VYIALPPLYKMSKGKGKKEEVAYAWTDGELEELRKQFGKGATLQRYKGLG EMNADQLWETTMNPETRTLIRVTIEDLARAERRVNVLMGDKVEPRRKWIED NVKFTLEEATVF E-site DNA1 5'- CATGAATGACTATGCACG-3' E-site DNA2 5'-CGTGCATAGTCATTCATG-3'

Topoisomerase IV core was formed by mixing ParC55 and ParE30 at an equimolar ratio in higher salt buffer (20 mM Tris-HCl, pH 7.5, 200 mM NaCl, 1 mM β-mercaptoethanol, 0.05% NaN_3_). The protein was then dialysed into lower salt buffer (20 mM Tris-HCl, pH 7.5, 100 mM NaCl, 1 mM β-mercaptoethanol, 0.05% NaN_3_) and the cleavage complexes were formed by mixing topo IV and E-site 18mer DNA at 1 : 1.2 molar ratio, respectively. Magnesium chloride and the drug of interest were added to final concentrations of 10 mM and 1 mM, respectively. The complexes were allowed to form by incubation at room temperature overnight.

Crystallization information is summarized in table 2. Crystals of the cleavage complexes were obtained by vapour diffusion using the sitting drop technique in MRC Wilden crystallization plates. Drops were formed at 600 : 400 nl ratio for complex and precipitant solution, respectively, using a Mosquito robot from TTP Labtech (www.ttplabtech.com). The crystallization was performed using a gradient grid varying pH from 6.0 to 7.0, NaCl from 100 to 140 mM and isopropanol from 4 to 7%. The rest of the crystallization cocktail was kept constant (i.e. 50 mM Na cacodylate, 2% Tacsimate; Hampton Research) [35]. Crystals of varying quality appeared stochastically throughout the gradients used and no clear indication of one strictly preferred crystallization condition was found within the boundaries of the grid employed. Significant prep-to-prep variation was also observed when different batches of the protein were used. Hence, it was found to be essential to scan a range of conditions each time the crystallization was performed. The best crystals were selected, briefly placed into a cryoprotectant solution (50 mM Na cacodylate, pH 6.5, 2% Tacsimate [35], 62.5 mM KCl, 7.5 mM MgCl_2_, 1 mM β-mercaptoethanol, 30% (v/v) MPD) and then frozen directly in liquid nitrogen.

**Table 2.** Crystallization.

method	vapour diffusion, sitting drop
plate type	MRC Wilden 96 wells plate
temperature (K)	295
protein concentration	4–5 mg ml^−1^
buffer composition of protein solution	20 mM Tris, pH 7.5, 100 mM NaCl, 1 mM β-mercaptoethanol, 0.05% NaN_3_
composition of reservoir solution	50 mM Na cacodylate, 4–7% 2-propanol, optimized mixture of salts (2% Tacsimate, 100–140 mM NaCl), pH 6.5
volume and ratio of drop	600 nl : 400 nl protein : reservoir
volume of reservoir	80 µl

### 2.3. Crystallization and data collection

Data collection and processing are summarized in table 3. Data collection was performed using the GDE software available on the I02 beamline at Diamond SLS, Oxfordshire. The programs XDS and XSCALE [36,37] were used for data reduction. Structure solution and refinement are summarized in table 4. The structures were solved with PHASER [38] as implemented in the CCP4 suite [39]. Our 3K9F structure was used as the starting protein model. Refinement was performed with PHENIX [40,41] using multiple rounds of coordinate and temperature factor refinement (employing TLS and secondary structure geometry restraints). Manual model fitting and correction was performed in WinCoot [42,43].

**Table 3.** Data collection and processing. Values for the outer shell are given in parentheses.

	ACHN-245	ACHN-454
diffraction source	Diamond beamline I02	Diamond beamline I02
wavelength (Å)	0.9795	0.9795
temperature (K)	100	100
detector	ADSC quantum 315 CCD	ADSC quantum 315 CCD
crystal-detector distance (mm)	391.900	391.926
rotation range per image (°)	0.50	0.25
total rotation range (°)	82.5	114.0
exposure time per image (s)	4.0	0.5
space group	*P*3_1_21	*P*3_1_21
*a*, *b*, *c* (Å)	157.83, 157.83, 210.71	157.86, 157.86, 210.78
*α*, *β*, *γ* (°)	90, 90, 120	90, 90, 120
mosaicity (°)	0.167	0.107
resolution range (Å)	41.819–3.43 (3.52–3.43)	41.828–3.24 (3.32–3.24)
total no. of reflections	206 644	337 161
no. of unique reflections	40 043	48 761
completeness (%)	97.1 (98.20)	99.9 (100.00)
redundancy	5.16 (5.22)	6.91 (7.13)
〈*I*/*σ*(*I*)〉	14.38 (6.37)^a^	19.16 (5.91)^a^
*R*_r.i.m._ (%)^b^	13.587 (44.3)	8.76 (41.1)
overall *B* factor from Wilson plot (Å^2^)	54.11	71.30

^a^The resolution cut-off used is based on the R-merge in the outer shell being less than 50% and *I*/sig(*I*) being above 1.5.

^b^Estimated *R*_r.i.m._ = *R*_merge_[*N*/(*N* − 1)]^1/2^, where *N* = data multiplicity.

**Table 4.** Structure solution and refinement. Values for the outer shell are given in parentheses.

	ACHN-245	ACHN-454
resolution range (Å)	41.819–3.430 (3.5157–3.4300)	41.828–3.240 (3.3061–3.2400)
completeness (%)	97.1	99.9
*σ* cut-off	*F* > 2.01*σ*(*F*)	*F* > 2.00*σ*(*F*)
no. of reflections, working set	40 016 (2722)	48 732 (2680)
no. of reflections, test set	2003 (134)	2446 (137)
final *R*_cryst_	0.154 (0.1825)	0.162 (0.2105)
final *R*_free_	0.195 (0.2386)	0.185 (0.2145)
no. of non-H atoms		
protein	10 388	10 339
nucleic acid	730	730
ion	6	6
ligand	54	54
water	24	14
total	11 202	11 143
r.m.s. deviations		
bonds (Å)	0.009	0.009
angles (°)	1.240	1.243
average *B* factors (Å^2^)		
protein	57.1	88.3
nucleic acid	65.6	99.2
ion	54.6	95.7
ligand	79.1	123.5
water	42.5	80.0
Ramachandran plot		
most favoured (%)	92	90
allowed (%)	6	8

Model quality was assessed using the in-built analysis tools of WinCoot (including geometry analysis and Ramachandran plot) as well as the verification tools provided by the PDB. Figures for this paper were generated using PyMOL [44], ChemDraw [45] and Corel Draw (www.coreldraw.com). The structure was verified using WinCoot and ProCheck [46].

### 2.4. Drug susceptibilities and topoisomerase assays

Bacterial susceptibility to drugs was determined by the broth microdilution assay following CLSI-recommended guidelines. Briefly, approximately 10^4^ CFU of the *S. pneumoniae* strains were inoculated in a final volume of 100 µl cation-adjusted Mueller–Hinton broth supplemented with lysed horse blood and twofold dilutions of the test compounds in 96-well plates. Plates were incubated at 35°C for 20–24 h. The MIC is the drug concentration at which no growth was seen when tested under these conditions.

Methods for assaying DNA gyrase and topoisomerase IV activity including DNA cleavage have been described previously [32]. Briefly, DNA cleavage assays were set-up using supercoiled pBR322 DNA (400 ng) as substrate. Full-length topo IV reconstituted by combining ParC (450 ng) and ParE (1 µg), or topo IV core domain ParE30-ParC55 fusion protein (400 ng), were incubated with DNA. Reaction buffer contained 40 mM Tris-HCl, pH 7.5, 6 mM MgCl_2_, 10 mM DTT, 200 mM potassium glutamate and 50 µg ml^−1^ BSA in a final volume of 20 µl with or without drug included. Samples were incubated at 37°C for 1 h followed by addition of 2 µl of 10% SDS to each reaction to induce DNA cleavage. After addition of proteinase K to 200 µg ml^−1^, incubation was continued at 42°C for 1 h to digest DNA-bound protein. Sample loading dye (5 µl) was added to each tube and DNA cleavage products were separated by gel electrophoresis in 1% agarose. DNA bands were stained with ethidium bromide and photographed under UV illumination. CC_25_ is the drug concentration used in the DNA cleavage assay that converted 25% of the supercoiled DNA substrate into linear DNA.

## 3. Results and discussion

**Figure 3.**
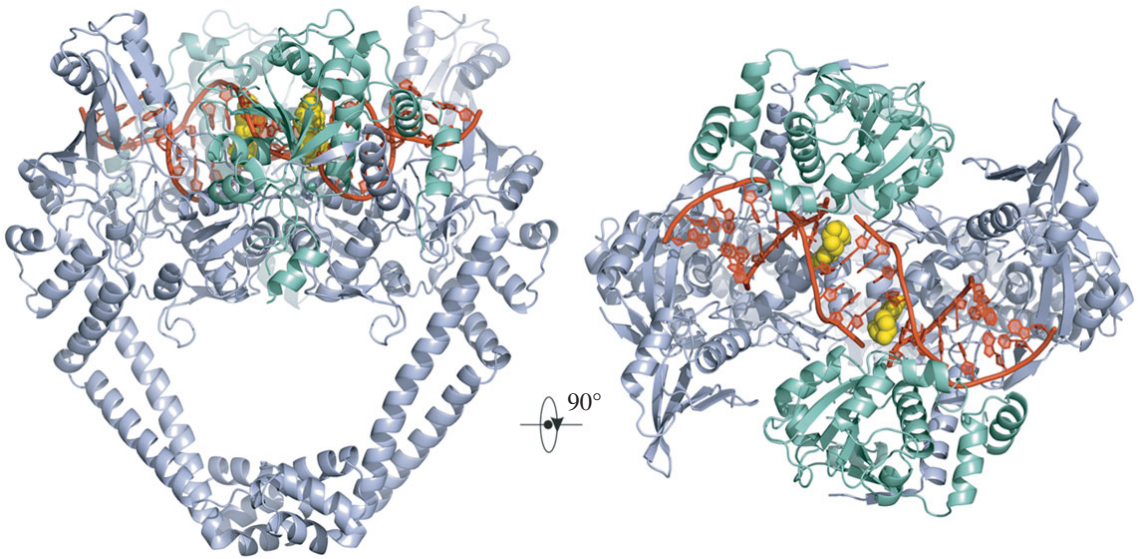
Orthogonal side and top views of the cleavage complex in cartoon representation respectively; ParC55 is in pale blue, ParE30 is in green-cyan, DNA is in red. Drug molecules are in yellow in van der Waals representation.

**Figure 4.**
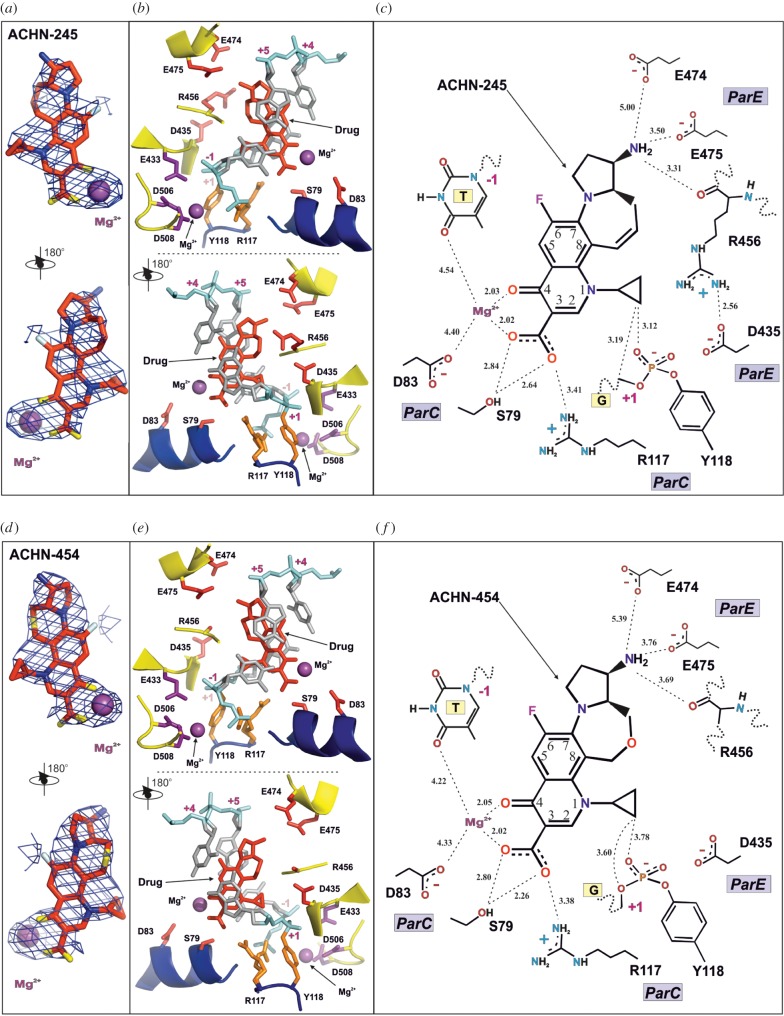
Detailed structural views of the binding sites for the quinolones ACHN-245 (*a*–*c*) and ACHN-454 (*d*–*f*). (*a*,*d*) Opposing views of the drug molecules with the chelated magnesium ions and the electron density from the 2*F*_obs_–*F*calc composite omit map contoured at the 1.5*σ* level. (*b*,*e*) Opposing views of the drug-binding site. ParC backbone is in blue, ParE backbone is in yellow, DNA backbone is in cyan, drug molecule is in red, magnesium ions are in purple, active site tyrosine (Y118) and arginine (R117) are in orange, DNA bases and sugars are in silver, active site magnesium-coordinating residues are in purple, residues responsible for drug resistance upon mutation are in red. (*c*,*f*) Schematic representation of the coordination of the drug in the active site. Important distances and likely hydrogen bonds are shown by dashed lines.

**Figure 5.**
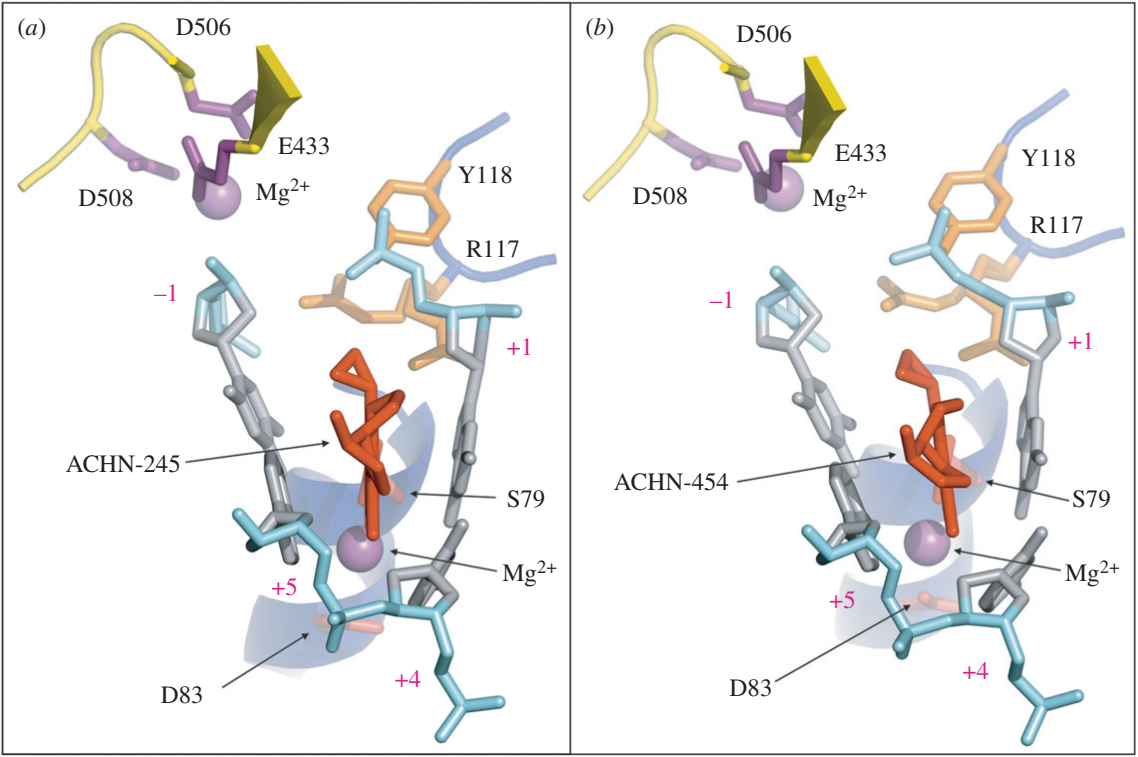
Top view on to the DNA cleft stabilized by the quinolone drugs ACHN-245 (*a*) and ACHN-454 (*b*).The ParC backbone is in blue, the ParE backbone is in yellow, DNA backbone is in cyan, drug molecule is in red, magnesium ions are in purple, active site tyrosine (Y118) and arginine (R117) are in orange, DNA bases and sugars are in silver, active site magnesium-coordinating residues are in purple, ParC S79 and D83 residues responsible for quinolone resistance upon mutation are in red.

**Figure 6.**
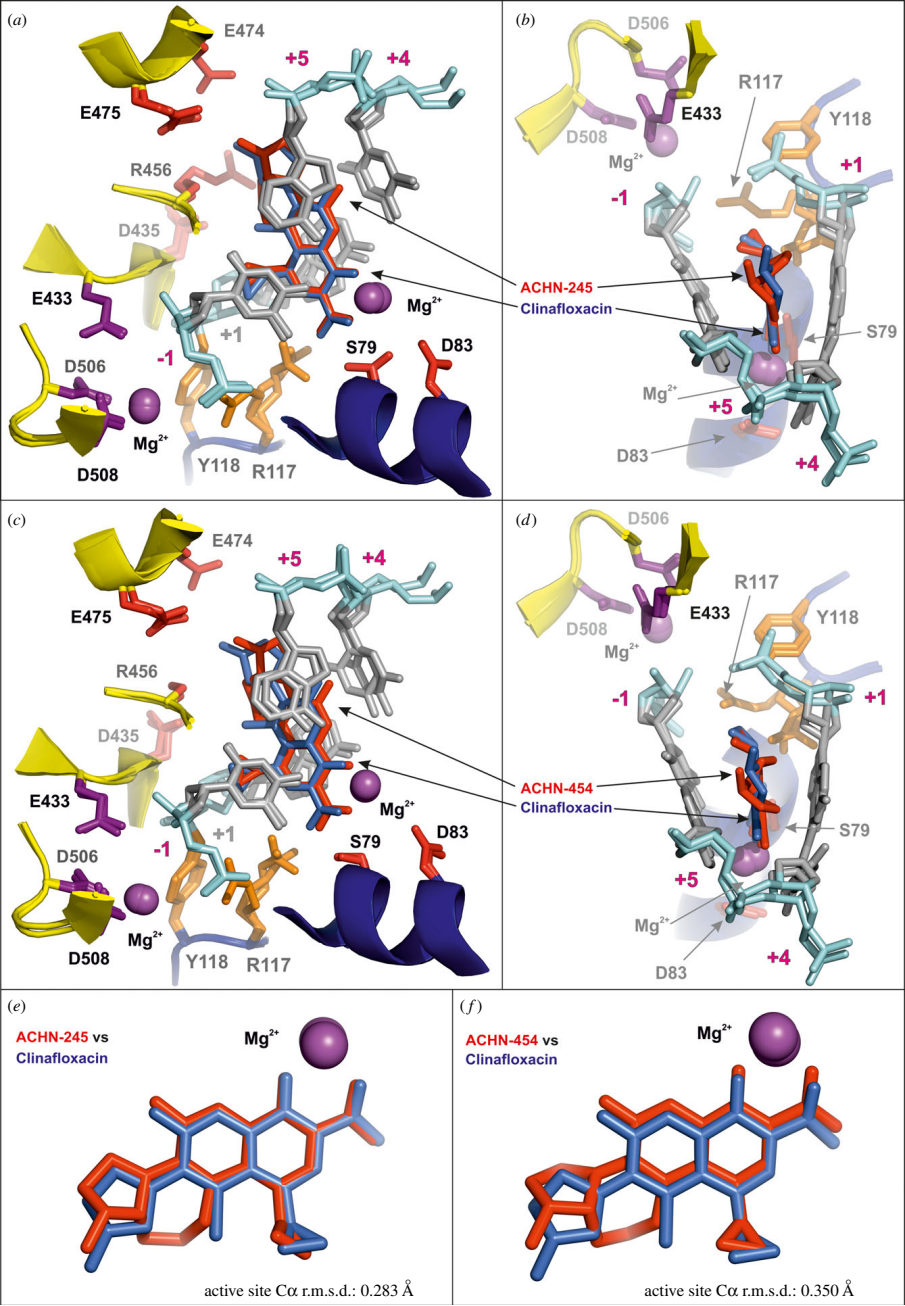
Comparison of cleavage complexes formed by 7,8-bridged fluoroquinolones and by clinafloxacin, their non-bridged counterpart. (*a*,*b*) Orthogonal views of the least-atom least-squares superposition of the ACHN-245 complex active site (4KPE) with that of the clinafloxacin complex (3RAD) determined at 3.35 Å resolution. (*c*,*d*) Orthogonal views of the least-atom least-squares superposition of the ACHN-454 complex active site (4KPF) with that of the clinafloxacin complex (3RAD). (*e*,*f*) Superposition of ACHN-245 (from 4KPE) (*e*) and of ACHN-454 (from 4KPF) (*f*) in each case with clinafloxacin (from 3RAD).

We have co-crystallized *S. pneumoniae* topoisomerase IV ParC–ParE breakage-reunion domain (ParC55, residues 1–490) and ParE TOPRIM domain (ParE30, residues 390–631) with an 18 bp DNA duplex (the E-site) stabilized by the novel drugs ACHN-245 and ACHN-454 synthesized by Achaogen [47]. The X-ray crystal structures of the complexes with ACHN-245 and ACHN-454 were determined at 3.43 and 3.24 Å, respectively, showing a closed ParC55 dimer flanked by two ParE30 monomers (figures 3–6). The macromolecular structure of this tetrameric complex is similar to that found for other *S. pneumoniae* topoisomerase–DNA–drug core complexes that we have previously reported [15–17]. We note that residues 6–30 of the N-terminal α-helix, α1 of the ParC subunit embrace the ParE subunit, pulling the ParE subunits close to either side of the ParC dimer (figure 3) [16]. This interaction, absent from our original ParC55 dimer structure [48,49], appears to be very important for ParC–ParE complex stability as deletion of the α-1 arm resulted in loss of DNA cleavage activity [48]. Figure 2*a* outlines the modular structure of the ParC and ParE proteins, mapping the positions of the TOPRIM metal binding domain, WHD (the winged helix domain) [50] and the TOWER regions of the protein subunits. This architecture has now been found in both topoisomerase IVs and gyrases from bacteria, and in topo IIα and topo IIβ from eukaryotes. The upper part of the topoisomerase complex consists of the E-subunit TOPRIM domain formed of four parallel β-sheets and surrounding α-helices. The WHD within the C-subunit together with the TOWER forms the U-groove-shaped protein region into which the G-gate DNA binds inducing a banana-shaped bend. The lower C-gate region (figure 3*a*) forms the region of the structure through which the T-segment DNA is envisaged to exit (see figure 1 illustrating the stages in the catalytic cycle) and is formed of a pair of two long α-helices terminated by a spanning short α-helix. Dimerization of the C domains at this point forms the 30 Å cavity, which will accommodate a B-DNA helix. The topoisomerase IV from *S. pneumoniae* is thought to follow the generic topoisomerase catalytic cycle shown in figure 1 involving putative intermediates 1–4, for which we have confirmation of intermediate 1 from our recent structure of the full complex (the holoenzyme less the CTD β-pinwheel domain) with the ATPase domains in the open conformation [3]. Moreover, a closed state with dimerized ATPase domains has been observed in a yeast topo II structure from Berger's group [51].

The G-gate DNA for our *S. pneumoniae* complexes stabilized by 7,8-bridged quinolones is the 18 bp E-site sequence, our notation for a DNA site that we first found in cleavage mapping studies of the *S. pneumoniae* chromosome [29,30]. It is clear from the presence of covalent DNA–protein links to ParC Y118 residues that the protein structures solved here (and earlier [15,16]) represent quinolone-stabilized cleavage complexes formed by turning over the topoisomerase IV tetramer bound to DNA. Interestingly, the E-site DNA sequence has subsequently been used in the successful co-crystallization of quinolone–DNA cleavage complexes of *Acinetobacter baumannii* topoisomerase IV [31] and *Mycobacterium tuberculosis* DNA gyrase [52], though without identification or reference to its origins in the *S. pneumoniae* system. It appears that the E-site is a versatile DNA substrate that permits the crystallization and structure solution of complexes formed with a variety of type II topoisomerases and cleavage-enhancing drugs.

Within the *S. pneumoniae* topoisomerase IV complex, a 7,8-bridged quinolone is hemi-intercalated into each DNA strand and stacked against the DNA bases at the cleavage site (positions −1 and +1 of the 4 bp staggered cut in the 18mer DNA) (figures 3–6) as found now in many other Gram-negative and Gram-positive topoisomerase IV and gyrase complexes. Figure 4*a,d* illustrates the electron density for the bound drugs in two views related by a 180° rotation showing the bound magnesium ion (purple sphere) within the electron density envelope coordinated between the carbonyl at position 4 on the quinolone ring and the carboxyl at position 3. How this magnesium ion makes further but longer interactions, now of around 4 Å in length with the thymidine base at position 1 and the ParC D83 side chain, is also shown (figure 4*b*,*c*,*e*,*f*). This is probably coordinated through a water molecule whose density we do not see but is theoretically confirmed by placing waters in a hexacoordinated system around the Mg^2+^. These interactions are the same for both ACHN-245 and ACHN-454 (figure 4*a–f*). The carboxyl at quinolone position 3, besides interacting with this magnesium ion, also makes interactions with ParC S79 and R117; the lengths of these interactions are similar for both drugs. Thus, the drug molecules are in close register to ParC S79 and D83 residues, two mutational hotspots to quinolone resistance [33,34,53,54]. The amino group on the 7-(3-aminopyrrolidin-1-yl) group makes key interactions with a cluster of two glutamates and an arginine (ParE E474, E475 and R456 in ParE) through a single oxygen of the carboxyl groups of the glutamates and the main chain carbonyl of R456 (figure 4*b,c*,*e*,*f*). The hydrogen atoms of the cyclopropyl substituents are within a 4 Å range of the two of the oxygens of the phosphotyrosine Y118. Note that a second Mg^2+^ ion at each drug-binding site coordinated by ParE E433, D506 and D508 interacts with the DNA phosphodiester group between −1 and −2 (figure 4*b*,*e*) and, through repositioning in the absence of drug, may be involved in DNA strand breakage-reunion.

Figure 5 shows an edge-on view and illustrates how the ACHN-245 and 454 drugs are hemi-intercalated into a wedge-shaped binding pocket, forming π–π* stacking interactions between the aromatic quinolone rings and the DNA bases. In both cases, the cyclopropyl group on N-1 of the quinolone is cocked out of the plane of the quinolone. Figure 5 presents the key disposition of the Mg^2+^ ions, with the Mg^2+^ coordinated to the carbonyl and carboxyl of the drug together with S79 and D83 side chains, located behind as part of an alpha helix. At the top left of figure 5*a,b*, the second Mg^2+^ is coordinated to the catalytic cluster of E433, D506, D508, a glutamate/aspartate triad which has been found in many eukaryotic, archaeal and prokaryotic topoisomerases as well as in many recombinases, nucleases and polymerases [55–59]. In figure 6 are shown two orthogonal views of how the drugs are sandwiched between the bases superposed with our highest resolution (2.9 Å) structure of the clinafloxacin cleavage complex. The stacking interactions are very similar, but the cycloheptyl group of these Achaogen compounds projects towards R456 and D435 (figure 6*a,c*; figure 4*c,f*) and if modified could form a scaffold for substituents which could span towards these and other residues and impart further contacts. The cyclogroup of the Achaogen compounds projects towards the van der Waals cavity formed by the sugars of the DNA about the semi-intercalation and the only protein side chain making a close approach is E475 (figure 6*a*,*c*). In figure 6*e*,*f* are shown the superpositions of the Achaogen compounds with clinafloxacin in the complex 3RAD where the 7-group is free to rotate in contrast with its locked conformation in the Achaogen compounds.

The disposition of the quinolone drugs in the topoisomerase binding site is very similar to that of levofloxacin, and clinafloxacin, and the ring structure gives hindered rotation to the 7-(3-aminopyrrolidin-1-yl) moiety and maintains key interactions with E474, E475 and R456. The disposition of the cycloheptyl group shows how with further chemical elaboration of this scaffold, further interactions with neighbouring residues in the binding pocket may be formed.

Both antimicrobial susceptibility and enzyme-DNA cleavage assays reveal the 7,8-bridged fluoroquinolones ACHN-245 and ACHN-454 to be potent anti-pneumococcal agents. Table 5 presents the MIC data for the two compounds against quinolone-susceptible and quinolone-resistant *S. pneumoniae* strains. For comparison, data are included for levofloxacin, a widely used anti-pneumococcal quinolone, and for clinafloxacin, the experimental 8-chlorofluoroquinolone that bears the same 3-aminopyrrolidin-1-yl group at position 7 that is bridged to C-8 in the Achaogen compounds. Against the two quinolone-susceptible ATCC reference strains, the two ACHN compounds exhibited MICs of 0.03–0.06 mg l^−1^ which is comparable to or marginally better than clinafloxacin (0.06 mg l^−1^) and much lower than the MICs for levofloxacin, typically 0.5–1 mg l^−1^ (table 5). Clearly, chemical bridging of the 7 and 8 positions in the ACHN compounds does not compromise anti-pneumococcal activity.

**Table 5.** Anti-pneumococcal activity of 7,8-bridged quinolones ACHN-245 and ACHN-454.

strain code	source	genotype	MIC (mg l^−1^)			
			levo floxacin	clinafloxacin	ACHN-245	ACHN-454
ASPN002	ATCC	wild-type	1	0.06	0.03	0.06
ASPN009	ATCC	wild-type	0.5	0.06	0.03	0.06
ASPN1010	clinical isolate, Mount Sinai (Toronto, Canada)	*gyrA*(S81Y)	2	0.5	0.25	0.25
ASPN1011	clinical isolate, Mount Sinai (Toronto, Canada)	*gyrA*(S81Y); *parC*(D83N)	8	0.5	0.5	0.5
ASPN1009	clinical isolate, Mount Sinai (Toronto, Canada)	*gyrA*(E85K); *parC*(S79Y); *parE*(I460V)	32	1	0.5	1
ASPN1027	clinical isolate, Mount Sinai (Toronto, Canada)	*gyrA*(S81F), *parC*(S79F, K137N)	32	2	1	1

Compared with wild-type strains, clinical isolates harbouring double quinolone-resistance mutations in gyrase and topoisomerase IV e.g. ParC79F and GyrA81F exhibited an 8–16-fold increase in MIC for ACHN-245 and ACHN-454 to 0.25–1 mg l^−1^, similar to the clinafloxacin MICs (table 5 and reported earlier in [60]). Thus, although quinolone-resistant strains have elevated MICs for ACHN-245 and ACHN-454, these MICs are very much lower than the typical levofloxacin MIC values of 32 mg l^−1^ (table 5). Evidently, as with clinafloxacin, the greater potency of the 7,8-bridged compounds extends to quinolone-resistant strains.

**Figure 7.**
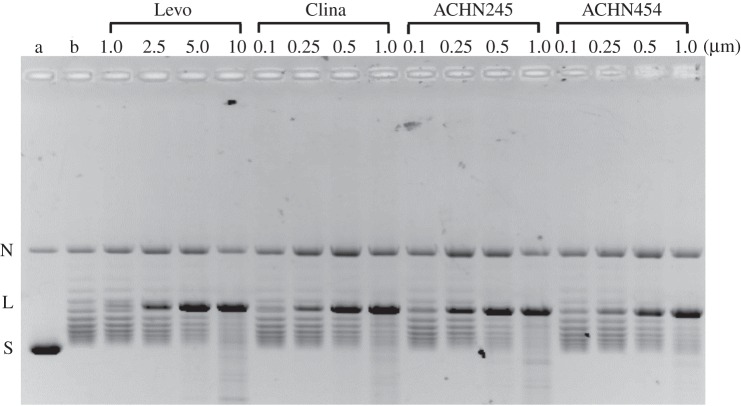
7,8-bridged fluoroquinolones are potent mediators of DNA cleavage by *Streptococcus pneumoniae* topoisomerase IV. Supercoiled plasmid pBR322 DNA (400 ng) was incubated with *S. pneumoniae* ParE30-ParC55 fusion protein (400 ng) in the absence or presence of levofloxacin (Levo), clinafloxacin (Clina), or the 7,8-bridged fluoroquinolones ACHN-245 and ACHN-454 at the indicated concentrations. After incubation at 37°C, samples were treated with SDS and proteinase K to remove covalently linked protein and the DNA products were examined by gel electrophoresis in 1% agarose, as described in the Material and methods. Lane a, supercoiled pBR322 DNA; lane b, DNA plus topo IV protein (no drug); N, L and S denote nicked, linear and supercoiled pBR322, respectively.

**Figure 8.**
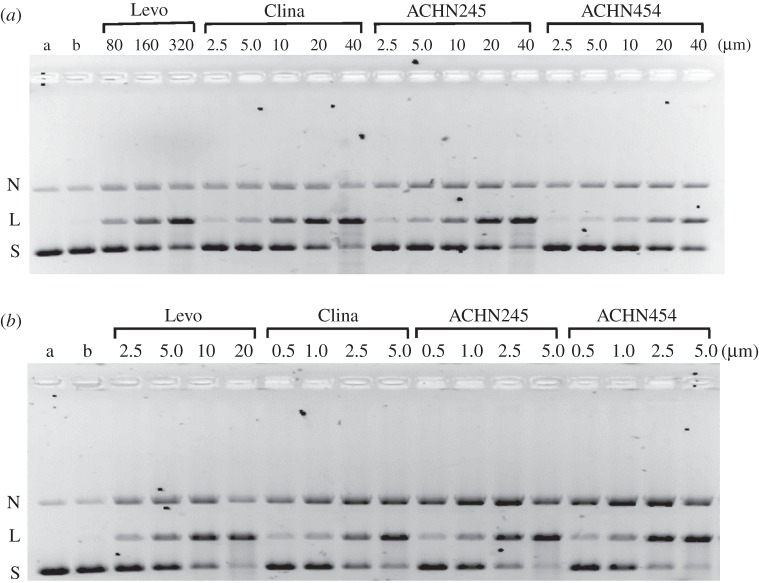
ACHN-245 and ACHN-454 promote DNA cleavage by fluoroquinolone-resistant (ParC79F) topoisomerase IV (*a*) and by dione-resistant (ParE475A) topoisomerase IV (*b*) from *Streptococcus pneumoniae*. Supercoiled plasmid pBR322 (400 ng) was incubated with full-length topo IV reconstituted from 450 ng ParC79F and 1 µg ParE (*a*) and 450 ng ParC and 1 µg ParE475A (*b*). DNA cleavage was induced and DNA products were analysed as described in the figure 7 legend. Lanes *a*, supercoiled pBR322; lanes *b*, DNA plus mutant topoisomerase IV enzyme and no drug.

In complementary biochemical experiments, we examined drug-induced DNA cleavage mediated by purified wild-type *S. pneumoniae* topoisomerase IV and by its ParC79F and ParE475A mutants that exhibit resistance to quinolones and to antibacterial quinazolinediones, respectively (figures 7 and 8) [27]. The trapping of a topoisomerase–DNA cleavage complex is thought to be the seminal event in quinolone and quinazolinedione action that underlies their cytotoxic action [27]. In studies of the wild-type enzyme, we used the topo IV core domain—a ParE30-ParC55 fusion protein—that carries all the determinants necessary for DNA cleavage. The protein was incubated with supercoiled pBR322 DNA in the absence and presence of drug, and following addition of SDS and proteinase K, the DNA products were separated and analysed by agarose gel electrophoresis (figure 7). As expected, in the absence of drug, the core enzyme converted supercoiled pBR322 into a ladder of relaxed DNA topoisomers (figure 7, lanes a and b), a well-documented activity of this truncated topoisomerase IV complex not seen for the full-length holoenzyme (figure 8) [32]. Inclusion of drug led to the formation of linear DNA in a dose-dependent fashion (figure 7). Comparing the CC_25_ values (the drug concentration that converted 25% of the supercoiled DNA substrate to the linear form), it is clear that the two 7,8-bridged quinolones exhibited a similar potency to clinafloxacin, with CC_25_ of 0.25–0.5 µM, about 10-fold more active than levofloxacin (figure 7; table 6).

**Table 6.** Comparison of drug potencies of ACHN245, ACHN454 with levofloxacin and clinafloxacin in DNA cleavage assays mediated by *S. pneumoniae* topo IV. Results were average of three independent experiments.

proteins	CC_25_ (μM)^a^			
	levo	clina	ACHN245	ACHN454
ParE30-ParC55 (WT)	2.5–5	0.25–0.5	0.25–0.5	0.5
ParC79F/ParE	160–320	20	20	20–40
ParE475A/ParC	5–10	2.5	2.5	2.5

^a^CC_25_, the drug concentration resulting in 25% conversion of supercoiled pBR322 DNA into the linear form.

Using full-length topoisomerase IV complexes with mutant ParC or ParE subunits, we could investigate the roles of ParC S79 and ParE E475 residues in drug action (figure 8). We could show that DNA cleavage mediated by the 7,8-bridged quinolones, levofloxacin and clinafloxacin was in each case much less efficient for the topoisomerase IV S79F mutant, with CC_25_ values 40–80-fold higher than seen with the wt enzyme (figure 8*a*; table 6). However, topo IV with a ParE 475A mutation showed only a twofold increase in CC_25_ for levofloxacin (reported previously in [61]), but a 10–20-fold increase for clinafloxacin, ACHN245 and ACHN454 (figure 8*b*; table 6). These results indicate that both ParC S79 and ParE E475 residues play a role in binding clinafloxacin and the 7,8-bridged quinolones (figures 4*c,f* and 6).

Previously, we showed that the dione-resistant ParE475A topoisomerase IV exhibited a similar 10–20-fold increase in the CC_25_ for PD 0305970 [27]. We note that the ParE 475A mutation was selected with this quinazolinedione, which has a 3-aminopyrrolidin-1-yl group at position 7 closely similar to that present in clinafloxacin and the 7,8-bridged quinolones, but absent from levofloxacin. In conclusion, it may be that the presence of a 3-aminopyrroline side chain is a key factor in determining quinolone and dione interactions with ParE, and thereby the resistance profile. In any event, these biochemical experiments with mutant topoisomerase enzymes begin to map out potential drug–side chain interactions explored by the 7,8-bridged quinolones and by levofloxacin, a 1,8-bridged quinolone. It appears that the 7,8-bridging chemistry in the ACHN compounds does not reduce potency and suggests there is scope to explore further substitutions along the C1, C7 and C8 side of the quinolone that may enhance drug activity against resistant strains [61,62].

The ACHN-245 and ACHN-454 *S. pneumoniae* topoisomerase IV structures were deposited in the PDB with accession codes 4KPE and 4KPF, respectively.
